# Cigarette smoke-induced LKB1/AMPK pathway deficiency reduces EGFR TKI sensitivity in NSCLC

**DOI:** 10.1038/s41388-020-01597-1

**Published:** 2020-12-17

**Authors:** Fang-Ju Cheng, Chia-Hung Chen, Wen-Chen Tsai, Bo-Wei Wang, Meng-Chieh Yu, Te-Chun Hsia, Ya-Ling Wei, Yu-Chun Hsiao, Dai-Wei Hu, Chien-Yi Ho, Tzong-Shiun Li, Chun-Yi Wu, Wen-Yu Chou, Yung-Luen Yu, Chih-Hsin Tang, Chih-Yi Chen, Chuan-Mu Chen, Jennifer L. Hsu, Hsiao-Fan Chen, Yeh Chen, Chih-Yen Tu, Mien-Chie Hung, Wei-Chien Huang

**Affiliations:** 1 Graduate Institute of Basic Medical Science, China Medical University, Taichung, 404 Taiwan; 2 Department of Molecular and Cellular Oncology, The University of Texas MD Anderson Cancer Center, Houston, TX 77030 USA; 3 Graduate Institute of Biomedical Sciences, China Medical University, Taichung, 404 Taiwan; 4 Division of Pulmonary and Critical Care Medicine, Department of Internal Medicine, China Medical University Hospital, Taichung, 404 Taiwan; 5 School of Medicine, China Medical University, Taichung, 404 Taiwan; 6 Department of Respiratory Therapy, China Medical University, Taichung, 404 Taiwan; 7 Taiwan Clinical Trial Consortium for Lung Diseases (TCoC), Taichung, 404 Taiwan; 8 Department of Health Services Administration, China Medical University, Taichung, 406 Taiwan; 9 Center for Molecular Medicine, China Medical University Hospital, Taichung, 404 Taiwan; 10 Department of Biomedical Imaging and Radiological Science, China Medical University, Taichung, 404 Taiwan; 11 Division of Family Medicine, Physical Examination Center, and Department of Medical Research, China Medical University Hsinchu Hospital, Hsinchu, 302 Taiwan; 12 Department of Plastic Surgery, and Innovation Research Center, Show Chwan Memorial Hospital, Changhua, 500 Taiwan; 13 Department of Biomedical Imaging and Radiological Sciences, National Yang-Ming University, Taipei, 112 Taiwan; 14 The Ph.D. Program for Cancer Biology and Drug Discovery, China Medical University, Taichung, 404 Taiwan; 15 Institute of New Drug Development, China Medical University, Taichung, 404 Taiwan; 16 Drug Development Center, Research Center for Cancer Biology, China Medical University, Taichung, 404 Taiwan; 17 Department of Biotechnology, Asia University, Taichung, 413 Taiwan; 18 Chinese Medicine Research Center, China Medical University, Taichung, 404 Taiwan; 19 Institute of Medicine, Chung Shan Medical University, Taichung, 402 Taiwan; 20 Division of Thoracic Surgery, Department of Surgery, Chung Shan Medical University Hospital, Taichung, 402 Taiwan; 21 Department of Life Sciences and Ph.D. Program in Translational Medicine, National Chung Hsing University, Taichung, 402 Taiwan; 22 The IEGG and Animal Biotechnology Center, National Chung Hsing University, Taichung, 402 Taiwan

**Keywords:** Cancer metabolism, Non-small-cell lung cancer, Predictive markers

## Abstract

Smoker patients with non-small cell lung cancer (NSCLC) have poorer prognosis and survival than those without smoking history. However, the mechanisms underlying the low response rate of those patients to EGFR tyrosine kinase inhibitors (TKIs) are not well understood. Here we report that exposure to cigarette smoke extract enhances glycolysis and attenuates AMP-activated protein kinase (AMPK)-dependent inhibition of mTOR; this in turn reduces the sensitivity of NSCLC cells with wild-type EGFR (EGFR^WT^) to EGFR TKI by repressing expression of liver kinase B1 (LKB1), a master kinase of the AMPK subfamily, via CpG island methylation. In addition, LKB1 expression is correlated positively with sensitivity to TKI in patients with NSCLC. Moreover, combined treatment of EGFR TKI with AMPK activators synergistically increases EGFR TKI sensitivity. Collectively, the current study suggests that LKB1 may serve as a marker to predict EGFR TKI sensitivity in smokers with NSCLC carrying EGFR^WT^ and that the combination of EGFR TKI and AMPK activator may be a potentially effective therapeutic strategy against NSCLC with EGFR^WT^.

## Introduction

The epidermal growth factor receptor (EGFR) is one of the crucial therapeutic targets in non-small cell lung cancer (NSCLC). The leucine-to-arginine substitution at residue 858 (L858R) and in-frame exon 19 deletion make up about 90% of EGFR activating mutations and cause higher binding affinity of the ATP-binding site of EGFR to tyrosine kinase inhibitors (TKIs), thereby increasing the vulnerability of EGFR to inhibition of its kinase activity [1, 2]. EGFR TKI has been approved by the FDA as the standard of care for the first-line treatment of patients with metastatic NSCLC harboring EGFR activating mutations with favorable clinical response (80%) and better survival outcome compared with those patients with wild-type EGFR-expressing NSCLC (EGFR^WT^ NSCLC) [3]. Patients with EGFR^WT^ NSCLC also demonstrate response to EGFR TKI as second-line therapy albeit with a lower rate of 20–30% [4, 5]. However, EGFR activating mutations are detected in only about 29% of patients with NSCLC worldwide among which the highest (47%) and lowest (12%) frequencies have been observed in Asia-Pacific and Oceania regions, respectively [6]. Because EGFR TKI treatment is frequently overlooked in a large proportion of patients with EGFR^WT^ NSCLC, it is critical to identify a biomarker to select those who will better respond to the therapy.

The changes in the design and manufacture of cigarette filter have been documented to lead to a shift in the histology from SCC to adenocarcinoma subtypes of lung cancer due to higher exposure and sensitivity of peripheral lung cells to mutagens and carcinogens [7, 8]. The association between cigarette smoking and development of lung adenocarcinoma increased gradually, and exposure to cigarette smoke not only promotes cancer cell progression and stemness but also reduces the sensitivity of NSCLC cells to EGFR TKI through several potential mechanisms, including EGFR hyperactivation, c-MET overexpression, and ABCG2-depednent drug efflux [9–12]. Up to 70% of smoker patients with NSCLC express EGFR^WT^ protein [13] and also had shorter progression-free survival (PFS) than those with activating mutations or without smoking history in response to EGFR TKI treatment [10, 14]. These observations suggest that cigarette smoke may confer TKI resistance in EGFR^WT^ NSCLC.

Most cancer cells depend on aerobic glycolysis to maximize energy production and fuel their rapid growth and progression by utilizing more glucose as energy and carbon sources. A growing body of evidence suggests that reprogramming of bioenergy production is involved in the cigarette smoke-induced cancer development [15, 16]. However, it remains unclear whether reprogramming of aerobic glycolysis is involved in the cigarette smoke-induced EGFR TKI resistance. In this study, we sought to further our understanding of the EGFR TKI resistance mechanisms in cigarette smoke exposure to provide new therapeutic strategy for those patients.

## Results

### Increased glucose uptake in cigarette smoking-associated EGFR TKI resistance in NSCLC

**Fig. 1:**
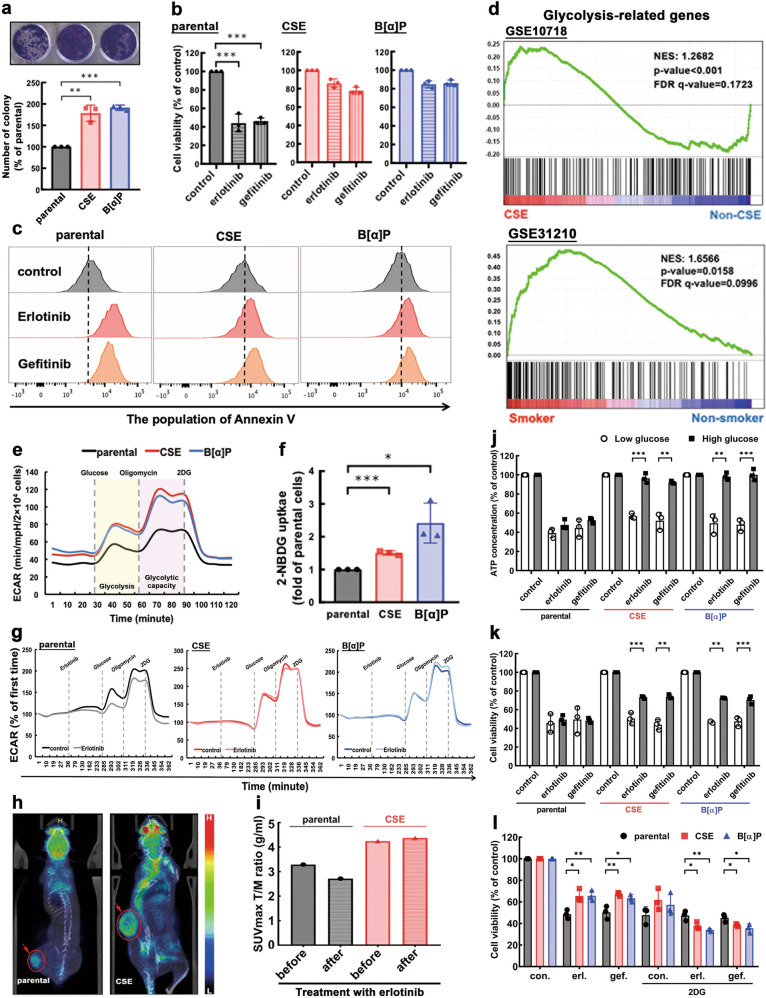
Exposure to CSE and B[α]P upregulated glucose metabolism to promote cell proliferation and EGFR TKI resistance. **a**–**c** CSE- and -B[α]P-selected H292 cells were subjected to colony formation assays followed by staining with 1% crystal violet (top) and quantitation (bottom) (**a**). The cell growth of H292 CSE- and B[α]P-selected cells treated with 1 μM of erlobinib and gefitinib for 3 days were measured by MTT assays (**b**) and FACS analysis (**c**), respectively. **d** The changes in the expression of glycolytic-related genes by CSE exposure (GSE10718 (top) or in smokers (GSE31210 (bottom)) were analyzed in GSEA. **e**–**g** CSE- and B[α]P-selected H292 cells was analyzed the glycolytic flux (**e**) and 2-NBDG uptake (**f**). The inhibitory effects of erlotinib treatment for 4 h on the glycolytic flux in CSE- and B[α]P-selected H292 cells were measured in Seahorse Analyzer (**g**). **h**, **i** The parental and CSE-selected H292 cells were injected into SCID mice followed by ^18^FDG uptake of tumor by microPET/CT analysis (**h**), and the inhibitory effect of erlotinib treatment (50 mg/kg) for 3 day on the maximum SUV in tumor (T) was measured and normalized to that in muscle (M) (**i**). **j**, **k** The ATP level (**j**) and cell viability (**k**) of CSE- and -B[α]P-selected H292 cells were treated with 1 μM of erlotinib and gefitinib under the conditions with different glucose concentrations for 3 days. **l** The cell viability of CSE- and B[α]P-selected H292 cells treated with 1 μM of erlotinib/gefitinib in the presence or absence of 5 mM 2DG were measured under 1 mM glucose culture condition for 2 day. Data are shown as mean ± SEM from experiments performed in triplicate. *p < 0.05; **p < 0.01; ***p < 0.001.

To address the mechanism underlying cigarette smoke-triggered EGFR TKI resistance in EGFR^WT^ NSCLC, H292 NSCLC cells, which express EGFR^WT^ and are sensitive to EGFR TKI, were subjected to long-term treatments with CSE or benzo[α]pyrene (B[α]P), a human carcinogen found in tobacco smoke, for at least 3 months to mimic the behavior of chronic smokers with NSCLC [12, 17]. As shown in Fig. 1a, the growth rate was significantly enhanced in CSE- and B[α]P-selected stable cells compared with the parental cells by colony assay. Erlotinib and gefitinib at 1 μM, the concentration within the clinically achievable steady-state plasma level [18, 19], were less effective in inducing cell killing (Fig. 1b), apoptosis (Fig. 1c), and migration suppression (Supplementary Fig. 2a) in the CSE- and B[α]P-selected H292 cells than in the control (parental) cells. The lower cell death of CSE-treated H322 cells in response to EGFR TKI was also found in cell viability assay (Supplementary Fig. 2b). These findings suggested that exposure to cigarette smoke renders EGFR^WT^ NSCLC cells more proliferative and resistant to EGFR TKIs.

To explore the potential mechanisms involved in cigarette smoke-induced resistance to EGFR TKI, gene set enrichment analysis (GSEA) was performed to profile CSE-related gene expression in 1094 patients with NSCLC, which did not include the EGFR status using the published gene sets [20–25]. The results indicated that pathways related to cell cycle, DNA damage, mTOR signaling, and reactive oxygen species were among the top twenty known to be enriched in response to cigarette smoke exposure (Supplementary Fig. 3a). Unexpectedly, the expression of genes related to glycolysis also increased significantly in both CSE-exposed lung alveolar cells (GSE10718) and smokers with NSCLC (GSE31210) (Fig. 1d). Although alteration in glycolysis is essential for fueling cancer cell proliferation, there is no evidence showing the association of dysregulation in glycolysis with CSE-triggered EGFR TKI resistance. The enhancement of glycolysis by CSE was further supported by the increase in basic ECAR in CSE- and B[α]P-selected H292 (Fig. 1e) and H322 (TKI-sensitive EGFR^WT^; Supplementary Fig. 3b) cells. The rate of glycolysis and glycolytic capacity were both enhanced by the CSE and B[α]P (Supplementary Fig. 3c). Exposure to CSE and B[α]P also increased glucose uptake (Fig. 1f), using 2-NBDG as an indicator, and ATP production (Supplementary Fig. 3d) in H292 cells. The CSE- and B[α]P-enhanced glycolytic flux (Fig. 1g and Supplementary Fig. 3e) and glucose uptake (Supplementary Fig. 3f) were not reduced when treated with EGFR TKI. Tumors developed in severe combined immunodeficient (SCID) mice implanted with CSE-treated H292 cells were bigger in size and exhibited stronger 18-fludeoxyglucose (^18^FDG) uptake compared with those from mice implanted with parental cells (Fig. 1h) as shown by microPET imaging. The standard uptake value (SUV) in the tumors (normalized to that in the muscle) [26] implied that CSE treatment dampened the suppressive effects of erlotinib on ^18^FDG uptake (Fig. 1i).

To further determine whether cigarette smoke promotes EGFR TKI resistance by upregulating glucose metabolism, parental H292 cells, and the CSE- and B[α]P-selected stable cells in culture media containing low or high glucose concentrations were treated with EGFR TKI. Both erlotinib and gefitinib suppressed ATP production (Fig. 1j) and cell viability (Fig. 1k) of the parental, and CSE- and B[α]P-selected cells under low glucose condition. In contrast, the inhibitory effects were reversed by high glucose concentrations in CSE- and B[α]P-selected cells but not parental cells. Treatment with 2-DG, a glucose analog which inhibits the glycolytic pathway, repressed CSE- and B[α]P-induced TKI resistance under low (Fig. 1l) but not high (Supplementary Fig. 3g) glucose conditions, suggesting that high glucose levels dampen the suppressive effects of 2-DG on TKI resistance. Together, these results indicated that cigarette smoke-induced cell proliferation and resistance to EGFR TKI are dependent on upregulation of glucose metabolism.

### Activation of AMPK by reducing glucose uptake and intracellular ATP contributes to sensitivity to EGFR TKI in NSCLC

**Fig. 2:**
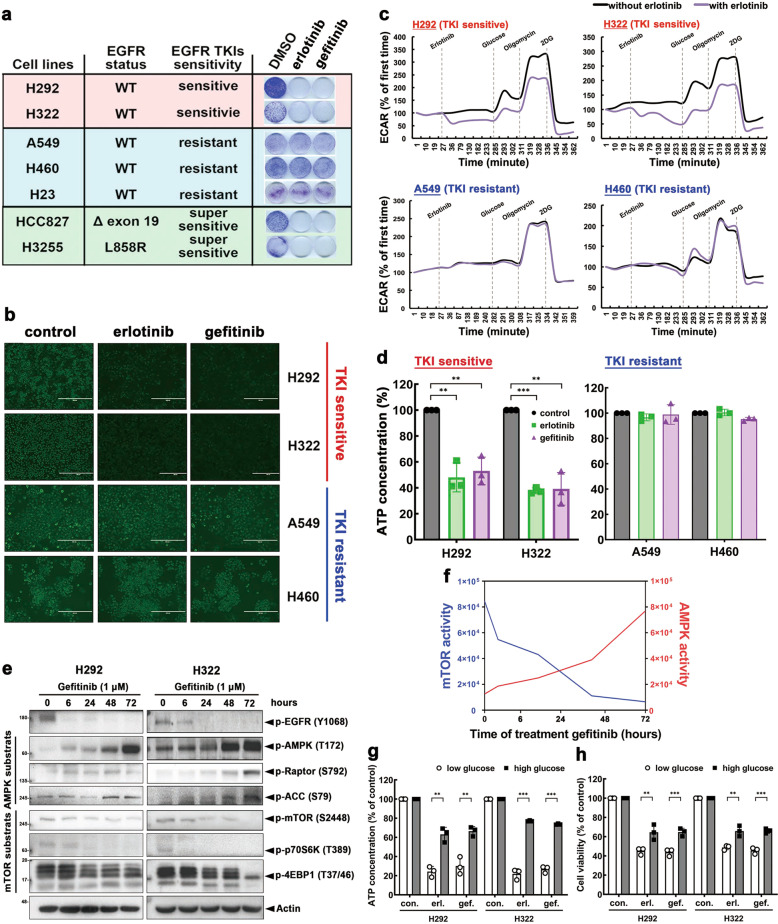
Activation of AMPK by reducing glucose uptake and intracellular ATP level contributes to the antiproliferative activity of EGFR TKI in NSCLC cells. **a** Lung cancer cell lines were treated with gefitinib or erlotinib for 7 days followed by 1% crystal violet staining. **b**–**d** H292, H322 (TKI sensitive) and A549, H460 (TKI resistant) treated with 1 μM of erlotinib or gefitinib for 3 days was examined in the level of 2-NBDG uptake (**b**), glycolytic flux (**c**), and ATP generation (**d**). Scale bars: 100 μm. **e**, **f** The effects of gefitinib treatment on the activity of EGFR/AMPK/mTOR signaling for the indicated time (**e**) and diagram of AMPK/mTOR activity (**f**) in various NSCLC cell lines. **g**, **h** The effects of glucose in culture media on ATP production (**g**) and viability (**h**) of various NSCLC cell lines by erlotinib (erl.) and gefitinib (gef.). Data are shown as mean ± SEM from experiments performed in triplicate. *P < 0.05; **P < 0.01; ***P < 0.001.

Given that dysregulation of glucose metabolism contributes to CSE-mediated resistance of EGFR^WT^ NSCLC to EGFR TKI (Fig. 1), we further expanded on the role of suppressing glucose metabolism on the antiproliferative activities of these drugs. First, we examined the effects of erlotinib and gefitinib on cell growth of various NSCLC cell lines harboring EGFR^WT^ (H292, H322, A549, H460, and H23) or EGFR activating mutations (HCC827 and H3255) by colony formation assay (Fig. 2a). As expected, EGFR mutant-expressing HCC827 and H3255 cells were highly sensitive to EGFR TKI (IC_50_ < 0.1 µM). Interestingly, EGFR^WT^-expressing cells can be divided into those sensitive (H292 and H322; IC_50_ < 3 µM) or resistant (A549, H460, and H23; IC_50_ > 10 µM) lines to erlotinib and gefitinib [27] (Fig. 2a). Both erlotinib and gefitinib attenuated 2-NBDG uptake (Fig. 2b), glycolytic flux (Fig. 2c), and ATP levels (Fig. 2d) in TKI-sensitive (H292 and H322) but not in TKI-resistant (A549 and H460) EGFR^WT^-expressing cells, suggesting an essential role of glycolysis inhibition in the sensitivity to EGFR TKI in EGFR^WT^ NSCLC cells.

Because EGFR TKI inhibited intracellular energy level by ATP depletion by blocking glucose uptake (Fig. 1), we thus examined the activation of AMP-activated protein kinase (AMPK), which is upregulated in response to ATP depletion and subsequently negatively regulates mTOR complex 1 to inhibit protein translation and tumor growth, in the presence of EGFR TKI. Treatment with gefitinib suppressed the kinase activity of EGFR after 6 h of treatment; suppressions of mTOR and mTORC1 substrate (p70S6K and 4EBP1) activities were observed after 24 h with simultaneous AMPK and AMPK substrate (Raptor and ACC) activations in TKI-sensitive H292 and H322 cells (Fig. 2e, f). However, neither erlotinib nor gefitinib diminished mTOR pathway or activated AMPK pathway in TKI-resistant A549, H23, and H460 cells even after 72 h of treatment (Supplementary Fig. 4a). These findings suggested that the increased AMPK activation as a result of reduced levels of intracellular glucose uptake and ATP contributes to EGFR TKI-induced mTOR suppression.

To determine whether dysregulation of glucose metabolism causes resistance to EGFR TKIs, lung cancer cells were cultured at high and low glucose conditions with or without TKI treatment. High glucose condition reversed EGFR TKI-induced ATP depletion (Fig. 2g) and cell growth suppression (Fig. 2h) in TKI-sensitive H292 and H322 cells. In contrast, no changes were observed in ATP level (Supplementary Fig. 4b) or cell growth (Supplementary Fig. 4c) by EGFR TKI in TKI-resistant A549 and H460 cells under either low or high glucose condition. These results suggested that reduction of intracellular ATP production and activation of AMPK due to inhibition of glycolysis have significant role in the sensitivity to EGFR TKI in NSCLC.

### LKB1 is required for EGFR TKI sensitivity in NSCLC

**Fig. 3:**
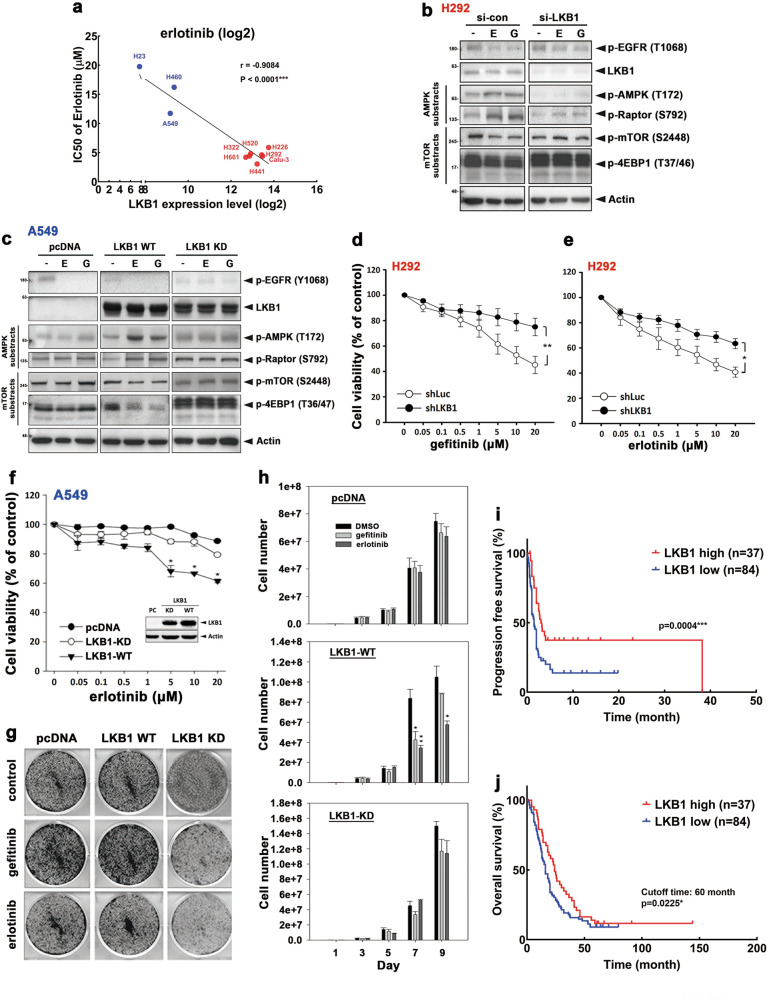
LKB1 expression positively correlates with clinical outcome of patients with NSCLC who received EGFR TKI. **a** The correlation between LKB1 protein level and IC50 of erlotinib in 10 EGFR^WT^-expressing lung cancer cell lines was analyzed. **b** Western blot analysis of H292 cells treated with 1 μM gefitinib or erlotinib for 3 days after transfection with LKB1 siRNA. **c** Western blot analysis of A549 cells transient transfected with WT or KD LKB1 cDNA and then treated with 1 μM erlotinib for 3 days. **d**, **e** H292 cells were treated with indicated concentrations of gefitinib (**d**) and erlotinib (**e**) for 3 days after transfection with LKB1 siRNA, and then subjected to MTT cell viability assay. **f**–**h** The cell viability (f), colony formation (**g**), and proliferation (**h**) of A549 stable transfectants expressing Flag-LKB1 WT or KD mutant in response to treatments with 1 μM gefitinib or erlotinib were determined by MTT, clonogenic, and cell counting assays, respectively. **i**, **j** The protein expression of LKB1 in tumor tissues from NSCLC patients was examined by IHC staining. Kaplan–Meier analysis of LKB1 expression and its correlation with progression-free survival (**i**) and overall survival (**j**). Data are shown as mean ± SEM from experiments performed in triplicate. *P < 0.05; **P < 0.01; ***P < 0.001.

Liver kinase B1 (LKB1), the upstream kinase of AMPK and a tumor suppressor gene, regulates intracellular energy homeostasis and activation of AMPK in the presence of high levels of intracellular AMP [28]. Next, we asked whether LKB1 expression is involved in determining EGFR TKI sensitivity in EGFR^WT^ NSCLC cell lines. Among the cell lines examined, LKB1 expression was detected in TKI-sensitive H226, H292, H322, H441, H520, H661, and Calu3 cells but absent in TKI-resistant A549, H460, and H23 cells (Supplementary Fig. 5a). LKB1 level is inversely correlated with the IC50 to erlotinib (Fig. 3a) and gefitinib (Supplementary Fig. 5b) in these cells. Next, to determine the role of LKB1 in TKI-induced AMPK activation and mTOR suppression, we knocked down LKB1 in H292 and H322 cells by using LKB1-specific siRNAs followed by treatment with gefitinib or erlotinib. Transient knockdown of LKB1 reduced AMPK and Raptor phosphorylation and reversed mTOR and 4EBP1 suppression without affecting EGFR inhibition by gefitinib and erlotinib in H292 (Fig. 3b) and H322 cells (Supplementary Fig. 6a). In contrast, overexpression of LKB1^WT^ but not its kinase-dead (KD) mutant enhanced the activations of AMPK and Raptor and inhibition of mTOR and 4EBP1 phosphorylation in A549 (Fig. 3c) and H460 (Supplementary Fig. 6b) cells in response to EGFR TKIs. Since both gefitinib and erlotinib induced AMPK activation by inhibiting glucose uptake and ATP level (Fig. 2), and phosphorylation of AMPK at T172 by LKB1 is known to enhance AMP-induced allosteric AMPK activation [29], the presence of LKB1 may be essential for EGFR TKI-elicited AMPK activation and mTOR suppression.

To further demonstrate the necessity of LKB1–AMPK activation for sensitivity to EGFR TKI, LKB1-knockdown cells were treated with EGFR TKI and subjected to MTT assays. Silencing LKB1 attenuated gefitinib- and erlotinib-mediated growth inhibition of H292 cells (Fig. 3d, e). We further examined whether exogenous expression of LKB1 can resensitize A549 cells to EGFR TKI. As shown in Fig. 3f, erlotinib treatment suppressed the viability of LKB1-deficient A549 cells reexpressing WT LKB1 but not the KD LKB1 mutant or control (pcDNA). Similarly, gefitinib and erlotinib also suppressed the colony formation of LKB1-deficent A549 cells reexpressing LKB1 WT but not LKB1 KD or control (Fig. 3g). The rate of cell growth of A549 cells was also attenuated by gefitinib and erlotinib when cells were re-introduced with LKB1 WT but not the KD mutant (Fig. 3h). Taken together, these results demonstrated that the LKB1–AMPK axis is essential for the sensitivity to EGFR TKI in EGFR^WT^ NSCLC cells.

Next, we asked whether LKB1 protein expression in tumor tissues correlates with the clinical outcome of patients with EGFR^WT^ NSCLC who received EGFR TKI treatments. Compared with patients with low expression of LKB1 (IHC staining score of 0–2+), those with high expression of LKB1 (IHC staining score of 3+) showed better response rates (partial response, PR; stable disease, SD) to EGFR TKI (Supplementary Table 1), and had better PFS (Fig. 3i) and overall survival (OS) (Fig. 3j). These results suggested that the LKB1 expression is a critical determinant for the sensitivity of EGFR^WT^ NSCLC to EGFR TKI.

### Exposure to CSE downregulates LKB1 expression via promoter DNA methylation and renders NSCLC resistant to EGFR TKI

**Fig. 4:**
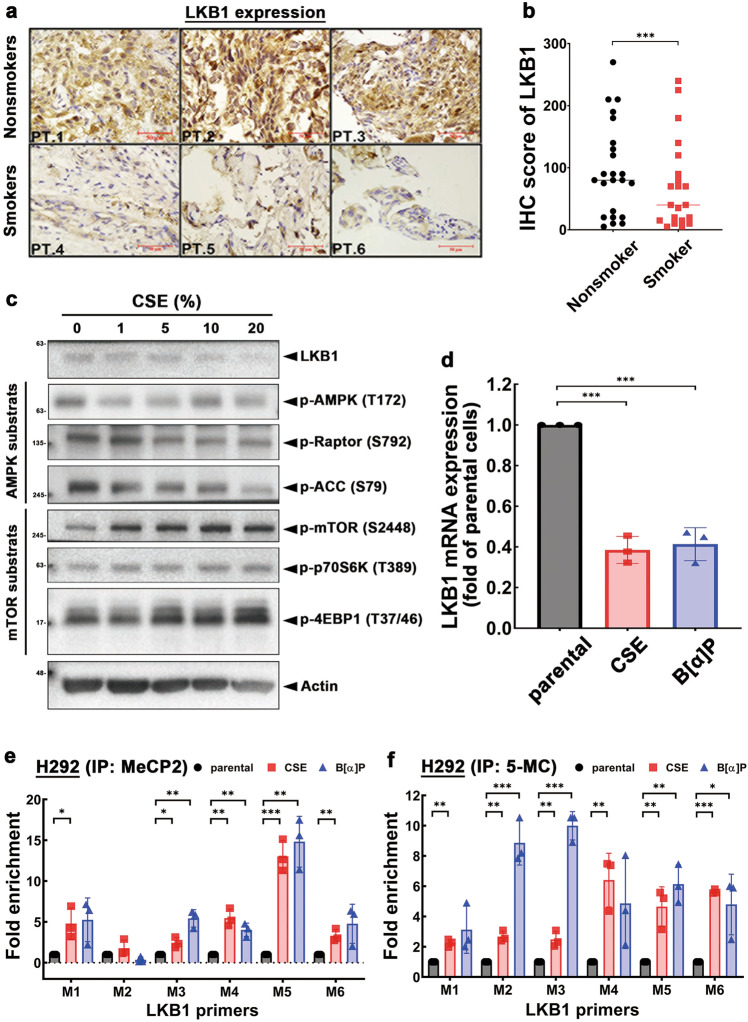
Cigarette smoke suppresses LKB1 expression via promoter methylation. **a** The protein expression of LKB1 in tumor tissues from NSCLC patients was analyzed by IHC staining. Scale bars: 50 μm. **b** The statistical data of IHC staining showed the correlation between expression of LKB1 and cigarette smoking in patients with EGFR^WT^ NSCLC. **c** Total lysates of H292 cells treated with various concentrations of CSE for 48 h followed by western blot analysis with indicated antibodies. **d** Analysis of mRNA expression of LKB1 from CSE- and B[α]P-treated H292 cells by RT-qPCR. **e**, **f** Total lysates of CSE and B[α]P H292 stable clones were subjected to ChIP-qPCR to identify the methylation status on the CpG island of *LKB1* promoter by anti-MeCP2 antibody (**e**) or anti-5-MC antibody (**f**). Data are shown as mean±SEM from experiments performed in triplicate. *P < 0.05; **P < 0.01; ***P < 0.001.

The *LKB1*-null mutation is frequently found in NSCLC tumors and associated with smoking behavior [30]. Because our data indicated LKB1 expression is positively associated with EGFR TKI sensitivity (Fig. 3), we hypothesized that cigarette smoke-mediated downregulation of LKB1 contributes to EGFR TKI resistance. Indeed, tumoral LKB1 expression was lower in smokers than in nonsmokers with NSCLC (Fig. 4a, b; Supplementary Table 1). Exposure to CSE reduced LKB1 protein expression and activation of AMPK signals and increased mTORC pathway in a dose-dependent manner in H292 cells (Fig. 4c). LKB1 mRNA expression was also suppressed by CSE and B[α]P in H292 cells (Fig. 4d). In former and current smokers with NSCLC, tumoral LKB1 mRNA expression was lower than that in never smokers with NSCLC in a published gene sets (GSE10072) with two probes (Supplementary Fig. 7a). These findings suggested that the LKB1 may be transcriptionally repressed by cigarette smoking.

Since DNA methyltransferase (DNMT) has been implicated in tobacco smoking-mediated promoter methylation and gene silence [31] and CSE dramatically suppressed LKB1 expression in H1299 cells (Supplementary Fig. 7b) which express higher DNMT isoforms [32], we next assessed whether cigarette smoke downregulates LKB1 transcription through promoter methylation. Chromatin immunoprecipitation with anti-5-methylcytosine (5-MC) and anti-methyl-CpG-binding protein 2 (MeCP2) antibodies, which recognized methylated DNA and the important reader of methylated DNA respectively, was performed followed by quantitative real-time PCR with six different primers targeting the CpG island on the *LKB1* promoter (illustrated in Supplementary Fig. 7c). Chronic CSE and B[α]P exposure significantly increased the levels of all 6 regions of the *LKB1* promoter in both anti-MeCP2 (Fig. 4e) and anti-5-MC (Fig. 4f) immunoprecipitates. These results suggested that cigarette smoke-downregulated LKB1 expression likely occurs via promoter DNA methylation and contributes to EGFR TKI resistance in NSCLC.

### AMPK activator metformin synergizes the antiproliferative activities of EGFR TKI in NSCLC

**Fig. 5:**
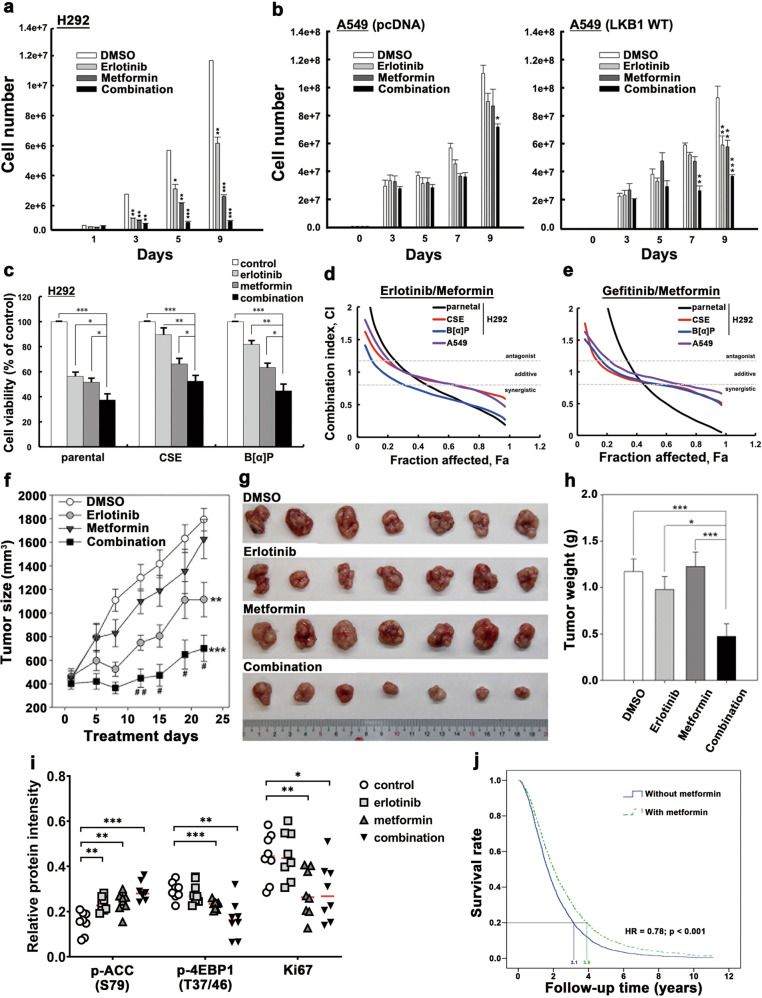
AMPK activator synergizes the antiproliferative activity of EGFR TKI in vitro and in vivo. **a**, **b** The cell counting assay of H292 (**a**) and A549 pcDNA and LKB1 WT (**b**) treated with erlotinib, metformin or their combination for the indicated days. **c**–**e** CSE- and B[α]P-selected stable H292 cells treated with erlotinib and metformin were subjected to MTT assays (**c**). The combination index of CSE- and B[α]P-treated H292 cells in response to erlotinib plus metformin (**d**) or gefitinib plus metformin (**e**) was determined by using CompuSyn. **f**–**i** H292 cells were inoculated subcutaneously into NOD-SCID mice followed by treatment with erlotinib, metformin, or the combination for the indicated days. The tumor size (**f**, **g**) and weight (**h**) were measured. Phosphorylation of ACC and 4EBP1, and expression of Ki67 in these tissue sections were quantified from IHC staining results (**i**). *n* = 7 mice for each group. **j** Overall survival curve of the metformin and non-metformin cohort of patients with NSCLC and T2DM who received TKI treatment. Data are shown as mean ± SEM from experiments performed in triplicate. *P < 0.05; **P < 0.01; ***P < 0.001.

On the basis of the above results showing that inactivation of the LKB1–AMPK axis contributes to EGFR TKI resistance in EGFR^WT^ NSCLC, we sought to explore potential approaches to overcome resistance. To date, there are no pharmacological activators of LKB1; however, metformin, an FDA-approved drug for T2DM, is a pharmacological activator of AMPK, and thus we asked whether metformin can enhance the antiproliferative activity of EGFR TKI to overcome resistance. To this end, we co-treated cells with metformin and showed that it enhanced the inhibitory effects of erlotinib (Fig. 5a, b) and gefitinib (Supplementary Fig. 8a–c) on cell growth of the sensitive H292 and A549 (expressing LKB1 WT stable cells) cells, but not the resistant A549 control cells. In addition, metformin remains able to induce AMPK activation in CSE- and B[α]P-selected clones (Supplementary Fig. 8d), and the suppressive effects of metformin plus erlotinib (Fig. 5c) was observed in these clones (the combination index (CI) shown in Fig. 5d, e). Combined treatment with mTORC1 inhibitor everolimus also showed similar effect (Supplementary Fig. 8e), suggesting that the enhancement of AMPK activity or inhibition of mTORC1 activity are potential strategies to enhance the antiproliferation of EGFR TKI in EGFR^WT^ NSCLC.

To further validate the inhibitory effects of metformin on the antiproliferative activity of EGFR TKI in vivo, SCID mice were implanted with H292 cells. After the tumors were established, vehicle, erlotinib, metformin, or the combination of erlotinib and metformin was administered to mice for the indicated days to monitor tumor size and weight. Compared with erlotinib or metformin alone, their combination dramatically reduced tumor size (Fig. 5f, g) and tumor weight (Fig. 5h). The erlotinib and metformin combination also enhanced AMPK-dependent phosphorylation of acetyl-coA carboxylase (ACC), a major downstream effector of AMPK in lipid metabolism, and attenuated mTOR-mediated 4EBP1 phosphorylation and Ki67 cell proliferation marker in the tumor tissues (Fig. 5i and Supplementary Fig. 8f). These results indicated that co-treatment with AMPK activator can synergize the antiproliferative activity of and overcome resistant to EGFR TKI in vivo.

The results above prompted us to compare the clinical responses from NHIRD of Taiwan of patients who took metformin with EGFR TKI. To this end, we analyzed the incidence and hazard ratio for 21,778 patients with NSCLC and T2DM who received EGFR TKI treatment in a population-based cohort study using data from 2004 to 2014 (Supplementary Fig. 1). Of these patients, 1000 were enrolled in the non-metformin cohort and 3181 in the metformin cohort. In order to reduce bias in patient characteristics between two groups, we performed a propensity score matching with a ratio of 1:2 between patients with metformin and those without metformin (Supplementary Table 2). After we had performed a propensity score matching, this study conducted Cox proportional hazard model analyses to compare the relative risk of mortality between two groups. The results showed that a reduction of mortality (Supplementary Table 3) and better survival rate (Fig. 5j) were observed in the metformin cohort (adjusted OR: 0.78; 95% CI: 0.69–0.88; *p* < 0.001). Together, the results lent further support to the benefits that the combination of EGFR TKI with AMPK activators may bring to patients with NSCLC.

## Discussion

The present study demonstrated the importance of the LKB1–AMPK axis in the therapeutic efficacy of EGFR TKI in patients with EGFR^WT^ NSCLC and a history of smoking. Through inhibition of glucose uptake, EGFR TKI triggered AMPK activation in an LKB1-dependent manner due to the reduction of ATP level to suppress mTOR signaling and tumor growth. Both in vitro and in vivo results revealed that NSCLC harboring LKB1^WT^ expression exhibited greater sensitivity and led to better clinical outcome in response to EGFR TKI.

Cancer cells predominantly utilize glycolysis to support the biosynthetic demands and fuel their proliferation. Targeting glycolytic enzymes has been shown to improve therapeutic response to anticancer agents [33], revealing the critical role of glycolysis in tumor progression and therapeutic resistance. Our results further indicated that exposure to CSE enhanced glucose uptake and aerobic glycolysis which in turn contributes to the growth of NSCLC, and this may in part account for the higher risk in tumor progression, failure to systemic therapy, and mortality for smokers with NSCLC. The expressions of two key glycolytic enzymes hexokinase II (HK-II) and pyruvate dehydrogenase kinase 2 (PKM2) are mediated by transcription factors hypoxia-inducible factor 1 (HIF-1) and tumor suppressor gene p53, respectively, and dysregulation of these two transcription factors by cigarette smoke [34, 35] represents a possible explanation for the enhanced glycolysis in CSE-treated NSCLC cells. In addition, upregulation of glycolysis also contributed to the CSE-mediated resistance of EGFR^WT^ NSCLC to EGFR TKI (Fig. 1). However, exposure to cigarette smoke has no bearing on TKI sensitivity of NSCLC cells harboring EGFR activating mutations [36], implying that dysregulation of genes or pathways particularly affected by CSE in EGFR^WT^ cells may explain the distinct impact of CSE on TKI sensitivity.

Somatic mutation(s) of *LKB1*, a tumor suppressor to upregulate AMPK-related catabolic and anabolic pathways, is viewed as an important cause of cancer development and poor prognosis in patients with NSCLC, hepatocarcinoma, and colorectal cancer [30, 37, 38]. Several studies have reported that EGFR mutations and *LKB1* deficiency are mutually exclusive in NSCLC patients [39, 40]. We discover that CSE exposure downregulated LKB1 expression via DNA promoter methylation to confer EGFR TKI resistance of EGFR^WT^ NSCLC. The enhancement of DNA methylation on gene promoters by tobacco exposure or nicotine treatment by activating DNA methyltransferase 1 (DNMT1) has been reported to cause abnormal target gene expression in bronchial epithelial cells and lung cancer cells [41, 42]. Aberrant CpG island methylation of *LKB1* has also been detected in primary lung tumor tissues and is associated with poor survival [43]. The expression of LKB1 was relatively lower in smokers with NSCLC even after cigarette smoking cessation following diagnosis of the disease compared with non smokers (Supplementary Fig. 7a). In addition, patients with NSCLC with loss function of LKB1 demonstrated poor response to EGFR TKI than those with LKB1^WT^ (Supplementary Table 1). Taken together, these findings supported the notion that cigarette smoke results in an irreversible dysfunction of LKB1 and confers EGFR TKI resistance in patients with NSCLC.

Metformin, an FDA-approved therapeutic agent for T2DM patients, is an AMPK activator that reduces the activity of complex I of the electron transport chain in the mitochondria due to decreased ATP generation and increased AMP and ADP content [44]. In addition to benefiting patients with diabetes mellitus, metformin also repressed cancer growth and prolonged the OS of those with advanced NSCLC [45, 46]. In this study, we further showed that metformin synergizes the antiproliferation activity of EGFR TKI in TKI-resistance and/or CSE-treated NSCLC cells by enhancing AMPK-mediated reduction of mTOR/4EBP1 and ACC pathways (Fig. 5). The data from NHIRD of Taiwan also demonstrated better survival rate in patients with NSCLC/T2DM who received EGFR TKI with metformin compared with those who did not receive metformin (Supplementary Table 3). Metformin has recently been reported to promote anticancer immunity via AMPK-dependent programmed death ligand-1 phosphorylation and degradation [47]. Importantly, NSCLC patients expressing activating EGFR mutation who were treated with both EGFR TKI and metformin had acquired good quality of life and experienced less side effects [46]. All these studies point to the notion that co-treatment with AMPK activators may be able to resensitize NSCLC cells bearing *LKB1*-null/EGFR^WT^ to EGFR TKI by suppressing glycolytic reprograming.

**Fig. 6:**
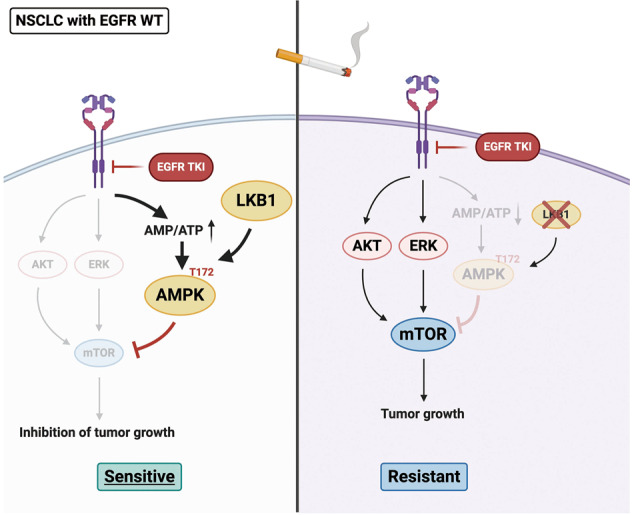
A proposed working model. LKB1 activation due to glycolysis inhibition is essential for the antiproliferative activity of EGFR TKI in EGFR^WT^ NSCLCs by activating AMPK to suppress mTORC1 function. However, cigarette smoke and its ingredient B[α]P represses LKB1 expression by enhancing its promoter CpG island methylation which renders NSCLC cells resistant to EGFR TKI. The addition of AMPK activator metformin can overcome EGFR TKI resistance in response to cigarette smoke exposure in patients with EGFR^WT^ NSCLC.

In summary, we demonstrated that EGFR TKI possesses antiproliferative activity in EGFR^WT^ NSCLC in an LKB1-dependent manner. By interfering with the glucose consumption, EGFR TKI reduced ATP production to activate the LKB1–AMPK axis, leading to suppression of mTOR-mediated cell proliferation. In addition, cigarette smoke reduced LKB1 expression, impairing its downstream AMPK signaling from suppressing mTOR activity (Fig. 6).

## Materials and methods

### Preparation of cigarette smoke extract (CSE) medium

Twenty-five commercial cigarettes (containing 0.8 mg nicotine and 10 mg tar per cigarette; Taiwan Tobacco & Liquor Corporation) were used to prepare 250 ml of culture medium. The pH value of CSE medium was adjusted to 7.4 followed by filtration with 0.22 μm filter to remove large particles. The stock of CSE medium is defined as 100% (1 cigarette per ml medium), and final concentration was calculated as following: [(ml CSE medium) ÷ (total ml medium) × 100].

### Measurement of extracellular acidification rate (ECAR)

NSCLC cells (2 × 10^4^ cells/well) were seeded in Seahorse XF24-well microplate (Agilent). After incubation for overnight, cells were cultured in assay medium without sodium bicarbonate and HEPES in a non-CO_2_ incubator at 37 °C for 1 h. Glucose oxidation was tested with consecutive additions of 1 μM erlotinib for 4 h, 10 mM glucose, 1 μM oligomycin, and 50 mM 2-DG (Seahorse XF Glycolytic Rate Assay Kit). ECAR was analyzed by Seahorse XF24 Analyzer (Agilent).

### 2-Deoxy-2-[(7-nitro-2,1,3-benzoxadiazol-4-yl)amino]-D-glucose (2-NBDG) utilization

Cells seeded in 6-well plates were treated with or without 1 μM gefitinib or erlotinib. Cell were washed with PBS three times and then incubated with glucose-free medium for 4 h. After wash with PBS for three times, the cells were incubated with PBS containing 2-NBDG (100 μM; Sigma-Aldrich) for 20 min at 37 °C and then subjected to flow cytometry analysis (BD Biosciences) or microscope image processing (ThermoFisher).

### Tumor xenograft mouse model

The 5-week-old female SCID mice (seven for each group), which bought from LASCO were injected with 1 × 10^6^ NCI-H292 cells suspended in 50 μl growth reduced Matrigel in right flank via a 22-gauge, 1.5-inch needle. Once tumor volume reached about 200 mm^3^, mice were treated with erlotinib 50 mg/kg/day or metformin 200 mg/kg/day by oral gavage. Tumor size was measured with calipers and tumor volume calculated using the formula: volume = width^2^ × length/2. After 10 weeks, the mice were sacrificed in a CO_2_ chamber and the tumors were collected. All the procedures of the animal experiments in study were approved by Institutional Animal Care and Use Committee of China Medical University (102-40-N) in accordance with NIH guidelines.

### Human lung tumor tissue specimens

The treatment naive tissue specimens from 121 patients with EGFR^WT^ NSCLC who have received EGFR TKI treatments (Supplementary Table 1) were obtained following the guidelines approved by Institute of Research Board Committee at China Medical University Hospital (DMR101-IRB1-120), and written informed consent was obtained from patients in all cases.

### Data source from Taiwan National Health Insurance Research Database (NHIRD)

NHIRD is a comprehensive health care database from the program of Nation Health Insurance that covers nearly the entire population in Taiwan. Data on the characteristics of patient, outpatient visit, and medicine administration was collected following the guidelines approved by Institute of Research Board Committee at China Medical University Hospital. The International Classification of Diseases, Ninth Revision, Clinical Modification (ICD-9-CM) codes were referred for the diagnosis of diabetes mellitus type 2 (T2DM) and NSCLCs. The patients with T2DM and NSCLCs diagnosed between 2004 and 2014 were collected from NHIRD. Patients who received EGFR TKI therapy, including erlotinib or gefitinib were included, while those diagnosed with multi- or other type of cancer, aged <20 years, T1DM, and incomplete information were excluded. Moreover, the patients with metformin usage more than 28 cumulative defined daily doses after suffering NSCLCs with EGFR TKI therapy were also enrolled (Supplementary Fig. 1).

### Statistical analysis

Data are shown as the mean ± standard error of the mean (SEM). A two-tailed *t* test was used for most comparisons, with *p* < 0.05 considered significant. PFS was calculated for the time period from the start of EGFR TKI (erlotinib or gefitinib) treatment to tumor recurrence. PFS and OS was analyzed by using Kaplan–Meier method and the statistical significance was analyzed by using Gehan–Breslow–Wilcoxon test. Chi-square test was used to determine the association between two categorical variables. A *P* value < 0.05 was considered statistically significant.

## Supplementary information

supplemental material
