# Supplementary material for: Cigarette smoke-induced LKB1/AMPK pathway deficiency reduces EGFR TKI sensitivity in NSCLC
Source: Oncogene. 2020 Dec 17;40(6):1162–75. doi: 10.1038/s41388-020-01597-1 (PMC7878190; doi:10.1038/s41388-020-01597-1)
Supplement: Supplementary file 1 — supplemental material [file 41388_2020_1597_MOESM1_ESM.docx]

Cigarette Smoke-induced LKB1/AMPK Pathway Deficiency Reduces EGFR TKI Sensitivity in NSCLC

Fang-Ju Cheng, Chia-Hung Chen, Wen-Chen Tsai, Bo-Wei Wang, Meng-Chieh Yu, Te-Chun Hsia, Ya-Ling Wei, Yu-Chun Hsiao, Dai-Wei Hu, Chien-Yi Ho, Tzong-Shiun Li, Chun-Yi Wu, Wen-Yu Chou, Yung-Luen Yu, Chih-Hsin Tang, Chih-Yi Chen, Chuan-Mu Chen, Jennifer L. Hsu, Hsiao-Fan Chen, Yeh Chen, Chih-Yen Tu, Mien-Chie Hung, Wei-Chien Huang

This PDF file includes:

Materials and Methods

Supplementary Fig 1~8

Supplementary Table 1~3

Materials and Methods

Cell lines and cell culture

The human lung cancer cell lines NSCLC NCI-H226, NCI-H292, NCI-H322, NCI-H23, NCI-H441, NCI-H460, NCI-H520, NCH-H661, HCC827, and H3255 cell lines were grown in RPMI 1640 medium, A549 cell line was cultured in DME/F12 medium, and Calu-3 cell line was maintained in Eagle’s Minimum Essential Medium (MEM) with 1 mM sodium pyruvate. All cells were cultured in the presence of 10% FBS, 1% streptomycin (100 μg/ml), 1.5g/L sodium bicarbonate, 1mM sodium pyruvate, and HEPES (25 mM) at 37°C in a humidified 5% CO_2_/95% incubator.

ATP level measurement

The intracellular ATP concentration was measured according to the manufacturer’s protocol. NSCLC cells (1 × 10^5^) in 6-well plates were treated with EGFR TKI for the indicated times and then subjected to ATP colorimetric/fluorometric assay (BioVision).

Western blot analysis

After the indicated treatments, NSCLC cells were lysed in RIPA buffer containing phosphatase and protease inhibitors. The protein lysates were separated by SDS-PAGE and were then transferred to PVDF membranes. The membranes were blocked in 5% BSA or 5% milk in TBST buffer (TBS with 0.1 % Tween 20), and were incubated with primary antibodies, which are LKB1 (#3047), phosphor-AMPK (#2535), AMPK (#5831), phosphor-EGFR (#3777), phosphor-Raptor (#89146), phosphor-mTOR (#5536), phosphor-p70S6K (#9234), phosphor-4EBP1 (#9459) from Cell Signaling as well as EGFR (sc373746) from Santa Cruz for overnight at 4 ºC followed by incubation with HRP-conjugated second antibody for 1 hour at room temperature. After washing with TBST buffer, the immunoreactive signals were visualized by using enhanced chemiluminescence with ECL reagent.

Isolation of total RNA, reverse transcription (RT), and quantitative RT-PCR assay

Total RNA was extracted from CSE and B[α]P-selected cells by using TRIzol reagent (Thermo Fisher Scientific) and then cDNA synthesis was performed with SuperScript first-strand synthesis system (Thermo Fisher Scientific) according to the manufacturer’s protocol. All samples from the product of RT were subjected to real-time PCR for examining the levels of LKB1 mRNA and 18s rRNA with specific Taqman probes (Thermo Fisher Scientific) and primers.

Chromatin immunoprecipitation (ChIP) assay

Total lysates from CSE- and B[α]P-treated H292 cells were isolated and fixed, and then digested with MNase (Thermo Scientific) followed by immunoprecipitation with the primary antibodies against 5-MC (Thermo Scientific; MA5-31473), MeCP2 (Cell Signaling; #3456) or IgG at 4 °C for overnight. The antibody-protein-DNA complex was pulled down with protein an agarose at room temperature for 4 hours and subjected to phenol/chloroform/isoamyl alcohol extraction (25:24:1; Invitrogen) to be used the template in SYBR FAST qPCR assays (KAPA Biosystems). Below are the primer sequences (5′ to 3′) for the methylated LKB1 promoter:

M1 region (­–11 to +202)

F: ATGGCAGGTTCAACCAACG; R: GCCGCCATCTTGTTTACCT

M2 region (–199 to –2)

F: AAGTTGGGCTCTCCAGGTGT; R: TCAGGGTCAACTTCGTCCTC;

M3 region (–1059 to –991)

F: CAGCTAAACAACCTGGCGCT; R: CAGGGGAAAGAGAAACGCTCA;

M4 region (–1299 to –1166)

F: CTTTCCCCTGGTCCAAGAGT; R’: CTCGGGAAGGGGGTCGTA;

M5 region (–1489 to –1253)

F: GTCTCCCCATGCCTGCTT, R: GTTGGTTGAACCTGCCATC;

M6 region (–1690 to –1480)

F: CAATGAGCGCGCTGTATCC, R: CGCCATCTTGTTTACCTCCCT.

Immunohistochemistry (IHC) Staining

Tumor tissue sections were dewaxed by xylene and rehydrated by different concentrations of ethanol. The mouse or patient tissue sections were incubated with phospho-ACC (1:300 dilution; Cell Signaling; #3661), phospho-4EBP1 (1:300 dilution; Cell Signaling; #9459), LKB1 (1:100 dilution; Santa Cruz; sc32245), or Ki67 (1:500 dilution; Thermo Scientific; MA5-14520) antibodies for overnight followed by the staining with a polymer HRP-conjugated secondary antibody for 30 min and by reaction with diaminobenzidine for 20 sec.





Supplementary Fig 1. A flow chart depicting the patient selection process





Supplementary Fig 2. Exposure to CSE and B[α]P reduces the sensitivity of NSCLC to EGFR TKIs. (a) The migration ability of CSE-/B[α]P-selected H292 stable cells treated with or without 1 μM erlotinib and gefitinib for 1 days was determined by wound healing analysis (left). Quantitation is shown right. (b) The viability inhibition of parental and CSE-selected H322 cells by 1μM erlotinib and gefitinib was examined and quantitated in clonogenic formation assay. Data are shown as mean ± SEM of experiments performed in triplicate. **P* < 0.05, ***P* < 0.01 and ****P* < 0.001.





Supplementary Fig 3. Exposure to CSE and B[α]P upregulates glucose metabolism to promote cell proliferation and EGFR TKI resistance. (a) The increased pathways in smoker NSCLC patients were analyzed in GSEA software from 5 databases (GSE31210, GSE29013, GSE43580, GSE50081, and GSE68465). (b and c) The glycolytic flux in CSE- and B[α]P-selected H322 stable cells was determined by Seahorse Analyzer (b). The glycolysis, glycolytic capacity, and glycolytic reversed in the CSE- and B[α]P-selected H292 (left) and H322 (right) cells were quantitated in (c). (d) The ATP level in CSE- and B[α]P-selected H292 stable cells was measured. (e) The effects of 1 μM erlotinib treatment for 4 hours on the ECAR in CSE- and B[α]P-selected H322 stable cells was determined by Seahorse Analyzer. (f) The 2-NBDG uptake of CSE- and B[α]P-selected H292 cells treated with 1 μM of erlotinib or gefitinib for 3 days was quantified by fluorescence microscopy. (g) The cell viability of CSE- and B[α]P-selected H292 stable cells in response to the combination of erlotinib and gefitinib with 5 mM 2DG under normal glucose culture condition was determined by MTT assays. Data are shown as mean ± SEM of experiments performed in triplicate. **P* < 0.05, ***P* < 0.01 and ****P* < 0.001.





Supplementary Fig 4. The effects of EGFR TKI on the AMPK/mTOR axis, ATP production, and cell viability in NSCLC cells. (a) WB analysis of the effects of 1μM erlotinib (Erl.) and gefitinib (Gef) on the activity of AMPK/mTOR pathways in A549, H23, and H460 cells. (b and c) The effect of erlotinib and gefitinib on the ATP concentration (b) and cell viability (c) of A549 and H460 cells under different glucose culture conditions. Data are shown as mean ± SEM of experiments performed in triplicate. **P* < 0.05, ***P* < 0.01 and ****P* < 0.001.





Supplementary Fig 5. The inverse correlation between LKB1 level and IC50 of EGFR TKI in NSCLC. (a) Whole cell lysates extracted from 10 EGFR^WT^-expressing lung cancer cell lines were subjected to Western blot analysis with indicated antibodies. (b) The correlation between LKB1 protein level and IC50 of gefitinib in these cell lines was analyzed.





Supplementary Fig 6. LKB1 mediates TKI-induced AMPK activation. (a) Western blot analysis of H322 cells treated with 1 μM gefitinib or erlotinib for 3 days after transfection with LKB1 siRNA. (b) Western blot analysis of H460 cells transient transfected with WT or KD LKB1 cDNA and then treated with 1 μM erlotinib for 3 days.





Supplementary Fig 7. The correlation between LKB1 downregulation and cigarette smoking in patients with NSCLC. (a) Quantitation of LKB1 mRNA expression in never, former, or current smokers with NSCLC a GEO database (GSE10072) with two individual probes. (b) Total lysates of H1229 cells treated with various concentrations of CSE for 48 hours followed by Western blot analysis with indicated antibodies. (c) A schematic showing the primer targeting sites on LKB1 promoter for qRT-PCR analysis shown in Figs 4E and 4F. **P* < 0.05.





Supplementary Fig 8. AMPK activator and mTORC inhibitor synergize the anti-proliferative activity of EGFR TKI. (a-c) A549 pcDNA (a), A549 LKB1 WT (b), and H292 (c) cells were treated with gefitinib, metformin, or their combination for indicated days and then subjected to cell counting assay. (d) Whole cell lysates extracted from parental, CSE- and B[a]P-selected H292 cells were subjected to Western blot analysis with indicated antibodies. (e) The cell viability of CSE- and B[α]P-selected H292 stable cells in response to the combinatory treatment with everolimus and metformin for 3 days was measured in MTT assays. The combination index was determined by using CompuSyn. (f) Phosphorylation of ACC and 4EBP1, and expression of Ki67 in these tissue sections were detected by IHC assay. Related to Fig 5I. Data are shown as mean ± SEM of experiments performed in triplicate. *P < 0.05, **P < 0.01 and ***P < 0.001.

Supplementary Table 1. Clinical characteristics and TKI response in NSCLC patients by LKB1 IHC status.

|  |  | LKB1 expression | |  |
| --- | --- | --- | --- | --- |
| *Characteristic* | Number | High (%) | Low (%) | *P* value |
| *Total (n)* | 121 | 37 (30.6) | 84 (69.4) |  |
| *Sex* |  |  |  |  |
| Male | 66 | 17 (45.9) | 49 (58.3) | 0.0894 |
| Female | 55 | 20 (54.1) | 35 (41.7) |  |
| *Age* |  |  |  |  |
| >55 | 86 | 30 (81.1) | 56 (66.7) | 0.024* |
| <55 | 35 | 7 (18.9) | 28 (33.3) |  |
| *Smoking* |  |  |  |  |
| Yes | 59 | 14 (37.8) | 45 (53.6) | 0.0232* |
| No | 62 | 23 (62.2) | 39 (46.4) |  |
| *Tumur size* |  |  |  |  |
| T1-T2 | 32 | 10 (27) | 22 (26.2) | 0.788 |
| T3-T4 | 71 | 21 (56.8) | 50 (59.5) |  |
| *Regional Lymph Nodes* |  |  |  |  |
| 0 | 19 | 7 (18.9) | 12 (14.3) | 0.0247* |
| 1 | 8 | 3 (8.1) | 5 (6) |  |
| 2 | 25 | 7 (18.9) | 18 (21.4) |  |
| 3 | 46 | 7 (18.9) | 39 (46.4) |  |
| *Pathological staging (AJCC)* |  |  |  |  |
| Stage I | 1 | 1 (2.7) | 0 (0) | 0.3118 |
| Stage II | 0 | 0 (0) | 0 (0) |  |
| Stage III | 11 | 4 (10.8) | 7 (8.3) |  |
| Stage IV | 100 | 32 (86.5) | 68 (81) |  |
| *TKI response* |  |  |  |  |
| PR + SD | 26 | 14 (37.8) | 12 (14.3) | 0.0001*** |
| PD | 95 | 23 (62.2) | 72 (85.7) |  |

**P* < 0.05, **P* < 0.01 and ****P* < 0.001

Supplementary Table 2. Descriptive statistics of patients with NSCLC who were or were not administered metformin.

| Variables | | Before matched | | | | | | | | | | | | | | After matched (1:2) | | | | | | | | | | | | | |
| --- | --- | --- | --- | --- | --- | --- | --- | --- | --- | --- | --- | --- | --- | --- | --- | --- | --- | --- | --- | --- | --- | --- | --- | --- | --- | --- | --- | --- | --- |
|  |  | Total | | | | Without Metformin | | | | With Metformin | | | | *P* value^a^ | | Total | | | | Without metformin | | | | With metformin | | | | *P* value^a^ | |
|  |  | N | | % | | N | | % | | N | | % | |  |  | N | | % | | N | | % | | N | | % | |  |  |
| Total number | | 4181 | | 100.00 | | 1000 | | 23.92 | | 3181 | | 76.08 | |  | | 2565 | | 100.00 | | 855 | | 33.33 | | 1710 | | 66.67 | |  | |
| Gender | |  | |  | |  | |  | |  | |  | | 0.636 | |  | |  | |  | |  | |  | |  | | 0.625 | |
|  | Female | 2036 | | 48.70 | | 494 | | 49.40 | | 1542 | | 48.48 | |  | | 1255 | | 48.93 | | 412 | | 48.19 | | 843 | | 49.30 | |  | |
|  | Male | 2145 | | 51.30 | | 506 | | 50.60 | | 1639 | | 51.52 | |  | | 1310 | | 51.07 | | 443 | | 51.81 | | 867 | | 50.70 | |  | |
| Age (years) | |  | |  | |  | |  | |  | |  | | < 0.001 | |  | |  | |  | |  | |  | |  | | 0.698 | |
|  | 20–54 | 495 | | 11.84 | | 96 | | 9.60 | | 399 | | 12.55 | |  | | 267 | | 10.41 | | 92 | | 10.76 | | 175 | | 10.23 | |  | |
|  | 55–64 | 1132 | | 27.07 | | 216 | | 21.60 | | 916 | | 28.80 | |  | | 643 | | 25.07 | | 207 | | 24.21 | | 436 | | 25.50 | |  | |
|  | 65–74 | 1476 | | 35.30 | | 325 | | 32.50 | | 1151 | | 36.18 | |  | | 934 | | 36.41 | | 305 | | 35.67 | | 629 | | 36.78 | |  | |
|  | ≧ 75 | 1078 | | 25.78 | | 363 | | 36.30 | | 715 | | 22.48 | |  | | 721 | | 28.11 | | 251 | | 29.36 | | 470 | | 27.49 | |  | |
|  | Mean ± SD | 67.24 ± 10.24 | | | | 69.62 ± 11.02 | | | | 66.49 ± 9.87 | | | | < 0.001 | | 67.94 ± 10.06 | | | | 68.23 ± 10.71 | | | | 67.79 ± 9.71 | | | | 0.318 | |
| CCI | | |  | |  | |  | |  | |  | | < 0.001 | |  | |  | |  | |  | |  | |  | | 0.915 | |  |
|  | 0 | 605 | | 14.47 | | 174 | | 17.40 | | 431 | | 13.55 | |  | | 417 | | 16.26 | | 145 | | 16.96 | | 272 | | 15.91 | |  | |
|  | 1 | 1465 | | 35.04 | | 287 | | 28.70 | | 1178 | | 37.03 | |  | | 815 | | 31.77 | | 267 | | 31.23 | | 548 | | 32.05 | |  | |
|  | 2 | 1173 | | 28.06 | | 264 | | 26.40 | | 909 | | 28.58 | |  | | 735 | | 28.65 | | 244 | | 28.54 | | 491 | | 28.71 | |  | |
|  | ≥ 3 | 938 | | 22.43 | | 275 | | 27.50 | | 663 | | 20.84 | |  | | 598 | | 23.31 | | 199 | | 23.27 | | 399 | | 23.33 | |  | |
| Stage | |  | |  | |  | |  | |  | |  | | <0.001 | |  | |  | |  | |  | |  | |  | |  | |
|  | 0 | 2 | | 0.05 | | 0 | | 0.00 | | 2 | | 0.06 | |  | |  | |  | |  | |  | |  | |  | | 0.290 | |
|  | I | 271 | | 6.48 | | 52 | | 5.20 | | 219 | | 6.88 | |  | | 126 | | 4.91 | | 51 | | 5.96 | | 75 | | 4.39 | |  | |
|  | II | 133 | | 3.18 | | 26 | | 2.60 | | 107 | | 3.36 | |  | | 69 | | 2.69 | | 25 | | 2.92 | | 44 | | 2.57 | |  | |
|  | III | 917 | | 21.93 | | 182 | | 18.20 | | 735 | | 23.11 | |  | | 515 | | 20.08 | | 175 | | 20.47 | | 340 | | 19.88 | |  | |
|  | IV | 2858 | | 68.36 | | 740 | | 74.00 | | 2118 | | 66.58 | |  | | 1855 | | 72.32 | | 604 | | 70.64 | | 1251 | | 73.16 | |  | |
| Diagnostic year | |  | |  | |  | |  | |  | |  | | <0.001 | |  | |  | |  | |  | |  | |  | | 1.000 | |
|  | 2004 | 87 | | 2.08 | | 17 | | 1.70 | | 70 | | 2.20 | |  | | 36 | | 1.40 | | 12 | | 1.40 | | 24 | | 1.40 | |  | |
|  | 2005 | 134 | | 3.20 | | 27 | | 2.70 | | 107 | | 3.36 | |  | | 75 | | 2.92 | | 25 | | 2.92 | | 50 | | 2.92 | |  | |
|  | 2006 | 190 | | 4.54 | | 50 | | 5.00 | | 140 | | 4.40 | |  | | 135 | | 5.26 | | 45 | | 5.26 | | 90 | | 5.26 | |  | |
|  | 2007 | 313 | | 7.49 | | 69 | | 6.90 | | 244 | | 7.67 | |  | | 189 | | 7.37 | | 63 | | 7.37 | | 126 | | 7.37 | |  | |
|  | 2008 | 369 | | 8.83 | | 68 | | 6.80 | | 301 | | 9.46 | |  | | 204 | | 7.95 | | 68 | | 7.95 | | 136 | | 7.95 | |  | |
|  | 2009 | 449 | | 10.74 | | 90 | | 9.00 | | 359 | | 11.29 | |  | | 252 | | 9.82 | | 84 | | 9.82 | | 168 | | 9.82 | |  | |
|  | 2010 | 424 | | 10.14 | | 94 | | 9.40 | | 330 | | 10.37 | |  | | 282 | | 10.99 | | 94 | | 10.99 | | 188 | | 10.99 | |  | |
|  | 2011 | 509 | | 12.17 | | 111 | | 11.10 | | 398 | | 12.51 | |  | | 333 | | 12.98 | | 111 | | 12.98 | | 222 | | 12.98 | |  | |
|  | 2012 | 612 | | 14.64 | | 156 | | 15.60 | | 456 | | 14.34 | |  | | 453 | | 17.66 | | 151 | | 17.66 | | 302 | | 17.66 | |  | |
|  | 2013 | 558 | | 13.35 | | 140 | | 14.00 | | 418 | | 13.14 | |  | | 309 | | 12.05 | | 103 | | 12.05 | | 206 | | 12.05 | |  | |
|  | 2014 | 536 | | 12.82 | | 178 | | 17.80 | | 358 | | 11.25 | |  | | 297 | | 11.58 | | 99 | | 11.58 | | 198 | | 11.58 | |  | |
| Health insurance (NT$) | |  | |  | |  | |  | |  | |  | | 0.332 | |  | |  | |  | |  | |  | |  | | 0.646 | |
|  | ≦ 17,280 | 143 | | 3.42 | | 31 | | 3.10 | | 112 | | 3.52 | |  | | 81 | | 3.16 | | 28 | | 3.27 | | 53 | | 3.10 | |  | |
|  | 17281-22800 | 1894 | | 45.30 | | 483 | | 48.30 | | 1411 | | 44.36 | |  | | 1191 | | 46.43 | | 407 | | 47.60 | | 784 | | 45.85 | |  | |
|  | 22281-28800 | 1021 | | 24.42 | | 235 | | 23.50 | | 786 | | 24.71 | |  | | 614 | | 23.94 | | 196 | | 22.92 | | 418 | | 24.44 | |  | |
|  | 28881-36300 | 323 | | 7.73 | | 77 | | 7.70 | | 246 | | 7.73 | |  | | 195 | | 7.60 | | 67 | | 7.84 | | 128 | | 7.49 | |  | |
|  | 36,301–45,800 | 387 | | 9.26 | | 84 | | 8.40 | | 303 | | 9.53 | |  | | 235 | | 9.16 | | 77 | | 9.01 | | 158 | | 9.24 | |  | |
|  | 45,801–57,800 | 179 | | 4.28 | | 44 | | 4.40 | | 135 | | 4.24 | |  | | 114 | | 4.44 | | 43 | | 5.03 | | 71 | | 4.15 | |  | |
|  | ≧ 57,801 | 234 | | 5.60 | | 46 | | 4.60 | | 188 | | 5.91 | |  | | 135 | | 5.26 | | 37 | | 4.33 | | 98 | | 5.73 | |  | |
| Hospital level | |  | |  | |  | |  | |  | |  | | 0.530 | |  | |  | |  | |  | |  | |  | | 0.977 | |
|  | Medical center | 2689 | | 64.31 | | 631 | | 63.10 | | 2058 | | 64.70 | |  | | 1666 | | 64.95 | | 556 | | 65.03 | | 1110 | | 64.91 | |  | |
|  | Regional hospital | 1428 | | 34.15 | | 351 | | 35.10 | | 1077 | | 33.86 | |  | | 855 | | 33.33 | | 285 | | 33.33 | | 570 | | 33.33 | |  | |
|  | District hospital | 64 | | 1.53 | | 18 | | 1.80 | | 46 | | 1.45 | |  | | 44 | | 1.72 | | 14 | | 1.64 | | 30 | | 1.75 | |  | |
| Hospital ownership | |  | |  | |  | |  | |  | |  | | 0.012 | |  | |  | |  | |  | |  | |  | | 0.064 | |
|  | Public | 1423 | | 34.03 | | 307 | | 30.70 | | 1116 | | 35.08 | |  | | 856 | | 33.37 | | 264 | | 30.88 | | 592 | | 34.62 | |  | |
|  | Private | 2758 | | 65.97 | | 693 | | 69.30 | | 2065 | | 64.92 | |  | | 1709 | | 66.63 | | 591 | | 69.12 | | 1118 | | 65.38 | |  | |
| NSCLC type | |  | |  | |  | |  | |  | |  | | 0.017 | |  | |  | |  | |  | |  | |  | | 0.701 | |
|  | Adenocarcinoma | 3707 | | 88.66 | | 908 | | 90.80 | | 2799 | | 87.99 | |  | | 2291 | | 89.32 | | 767 | | 89.71 | | 1524 | | 89.12 | |  | |
|  | Squamous cell carcinoma | 474 | | 11.34 | | 92 | | 9.20 | | 382 | | 12.01 | |  | | 274 | | 10.68 | | 88 | | 10.29 | | 186 | | 10.88 | |  | |
| Therapeutic combination | |  | |  | |  | |  | |  | |  | | <0.001 | |  | |  | |  | |  | |  | |  | | 0.172 | |
|  | None | 1079 | | 25.81 | | 309 | | 30.90 | | 770 | | 24.21 | |  | | 684 | | 26.67 | | 233 | | 27.25 | | 451 | | 26.37 | |  | |
|  | Surgery (S) | 248 | | 5.93 | | 47 | | 4.70 | | 201 | | 6.32 | |  | | 124 | | 4.83 | | 44 | | 5.15 | | 80 | | 4.68 | |  | |
|  | Chemotherapy (CT) | 1244 | | 29.75 | | 290 | | 29.00 | | 954 | | 29.99 | |  | | 802 | | 31.27 | | 255 | | 29.82 | | 547 | | 31.99 | |  | |
|  | Radiotherapy (RT) | 429 | | 10.26 | | 132 | | 13.20 | | 297 | | 9.34 | |  | | 298 | | 11.62 | | 119 | | 13.92 | | 179 | | 10.47 | |  | |
|  | S / CT | 229 | | 5.48 | | 37 | | 3.70 | | 192 | | 6.04 | |  | | 114 | | 4.44 | | 31 | | 3.63 | | 83 | | 4.85 | |  | |
|  | S / RT | 33 | | 0.79 | | 4 | | 0.40 | | 29 | | 0.91 | |  | | 17 | | 0.66 | | 4 | | 0.47 | | 13 | | 0.76 | |  | |
|  | CT / RT | 800 | | 19.13 | | 163 | | 16.30 | | 637 | | 20.03 | |  | | 472 | | 18.40 | | 153 | | 17.89 | | 319 | | 18.65 | |  | |
|  | S / CT / RT | 119 | | 2.85 | | 18 | | 1.80 | | 101 | | 3.18 | |  | | 54 | | 2.11 | | 16 | | 1.87 | | 38 | | 2.22 | |  | |

^a^ Chi-Square test

Supplementary Table 3. Adjusted hazard ratios of mortality in patients with NSCLC who were administered metformin.

| Variables | | Total | | Alive | | Death | | *P* value ^a^ | Adjusted HR | 95%CI | | *P* value ^b^ | |  |
| --- | --- | --- | --- | --- | --- | --- | --- | --- | --- | --- | --- | --- | --- | --- |
|  |  | N | % | N | % | N | % |  |  |  |  |  |  |  |
| Total number | | 2565 | 100 | 408 | 15.91 | 2157 | 84.09 |  |  |  |  | |  | |
| Metformin usage | |  |  |  |  |  |  | <0.001 |  |  |  | |  | |
|  | No | 855 | 33.33 | 117 | 13.68 | 738 | 86.32 |  |  |  |  | |  | |
|  | Yes | 1710 | 66.67 | 291 | 17.02 | 1419 | 82.98 |  | 0.78 | 0.69 | 0.88 | | <0.001 | |
| Gender | |  |  |  |  |  |  | <0.001 |  |  |  | |  | |
|  | Female | 1255 | 48.93 | 245 | 19.52 | 1010 | 80.48 |  |  |  |  | |  | |
|  | Male | 1310 | 51.07 | 163 | 12.44 | 1147 | 87.56 |  | 1.27 | 1.01 | 1.60 | | 0.041 | |
| Age (years) | |  |  |  |  |  |  | <0.001 |  |  |  | |  | |
|  | 20-54 | 267 | 10.41 | 38 | 14.23 | 229 | 85.77 |  |  |  |  | |  | |
|  | 55-64 | 643 | 25.07 | 107 | 16.64 | 536 | 83.36 |  | 0.88 | 0.54 | 1.43 | | 0.600 | |
|  | 65-74 | 934 | 36.41 | 159 | 17.02 | 775 | 82.98 |  | 1.06 | 0.67 | 1.66 | | 0.813 | |
|  | ≧75 | 721 | 28.11 | 104 | 14.42 | 617 | 85.58 |  | 1.27 | 0.79 | 2.04 | | 0.335 | |
| CCI | |  |  |  |  |  |  | <0.001 |  |  |  | |  | |
|  | 0 | 417 | 16.26 | 63 | 15.11 | 354 | 84.89 |  |  |  |  | |  | |
|  | 1 | 815 | 31.77 | 116 | 14.23 | 699 | 85.77 |  | 1.09 | 0.72 | 1.65 | | 0.690 | |
|  | 2 | 735 | 28.65 | 126 | 17.14 | 609 | 82.86 |  | 0.87 | 0.56 | 1.35 | | 0.536 | |
|  | ≥3 | 598 | 23.31 | 103 | 17.22 | 495 | 82.78 |  | 0.93 | 0.63 | 1.35 | | 0.693 | |
| Stage | |  |  |  |  |  |  | <0.001 |  |  |  | |  | |
|  | I | 126 | 4.91 | 40 | 31.75 | 86 | 68.25 |  |  |  |  | |  | |
|  | II | 69 | 2.69 | 15 | 21.74 | 54 | 78.26 |  | 3.61 | 1.62 | 8.08 | | 0.002 | |
|  | III | 515 | 20.08 | 87 | 16.89 | 428 | 83.11 |  | 4.60 | 2.41 | 8.81 | | <0.001 | |
|  | IV | 1855 | 72.32 | 266 | 14.34 | 1589 | 85.66 |  | 5.10 | 2.71 | 9.61 | | <0.001 | |

^a^ Log-rank test

^b^ Conditional cox proportional hazard model
